# Can Pediatricians Assess Exercise-Induced Bronchoconstriction From Post-exercise Videos?

**DOI:** 10.3389/fped.2019.00561

**Published:** 2020-01-23

**Authors:** N. Lammers, M. H. T. van Hoesel, M. G. J. Brusse-Keizer, J. van der Palen, R. Spenkelink-Visser, J. M. M. Driessen, B. J. Thio

**Affiliations:** ^1^Department of Pediatrics, Medisch Spectrum Twente, Enschede, Netherlands; ^2^Medical School Twente, Medisch Spectrum Twente, Enschede, Netherlands; ^3^Department of Research Methodology, Measurement and Data Analysis, University of Twente, Enschede, Netherlands; ^4^Department of Pediatrics, Gelre Ziekenhuizen, Apeldoorn, Netherlands; ^5^OCON Sport, Ziekenhuisgroep Twente, Hengelo, Netherlands; ^6^Department of Sportsmedicine, Tjongerschans Hospital, Heerenveen, Netherlands

**Keywords:** pediatric asthma, exercise-induced bronchoconstriction (EIB), children, videos, pediatricians

## Abstract

**Objective:** Exercise-induced bronchoconstriction (EIB) is a highly prevalent morbidity of childhood asthma and defined by a transient narrowing of the airways during or after physical exercise. An exercise challenge test (ECT) is the reference standard for the diagnosis of EIB. Video evaluation of EIB symptoms could be a practical alternative for the assessment of EIB. We studied the ability of pediatricians to assess EIB from post-exercise videos.

**Methods:** A clinical assessment was performed in 20 asthmatic children (mean age 11.6 years) and EIB was measured with a standardized ECT performed in cold, dry air. EIB was defined as a fall in forced expiratory volume in 1 s (FEV_1_) of ≥10% post-exercise. Children were filmed before and after exercise in frontal position and bare chested. The clinical assessment results and videos were shown to 20 pediatricians (mean experience 14.4 years). Each assessed EIB severity in 5 random children providing 100 assessments, scored on a continuous rating scale (0–10) and in severity classifications (no, mild, moderate, severe) using a scoring list including physical asthma symptoms. Correlations between predicted scores and objective scores were calculated with Spearman's rho and Cohen's Kappa. A generalized linear model was used to assess the relationship between physical symptoms and fall in FEV_1_.

**Results:** Median fall in FEV_1_ after exercise was 15.1% (IQR 1.2–65.1). Pediatricians detected EIB with a sensitivity of 78% (95% CI 66–87%) and a specificity of 40% (95% CI 27–55%). The positive predictive value for a pediatricians' diagnosis of EIB was 61% (95% CI 50–72%). The negative predictive value was 60% (95% CI 42–76%). The agreement between predicted EIB severity classifications and the validated classifications based on the ECT's, was fair [Kappa = 0.36 (95% CI 0.23–0.48)]. The correlation between predicted EIB severity scored on a continuous rating scale and fall in FEV_1_ after exercise was weak (r_s_ = 0.39, *p* < 0.001). Independent predictive variables for fall in FEV_1_ were wheezing (−11%), supraclavicular retractions (−8.4%) and a prolonged expiratory phase (−8.8%).

**Conclusion:** The ability of pediatricians to assess EIB from post-exercise videos is fair at best, implicating that standardized ECT's are still vital in the assessment of EIB.

## Introduction

Exercise-induced bronchoconstriction (EIB) is defined as a transient narrowing of the airways during or after physical exercise (1). Clinical symptoms include shortness of breath, chest tightness, wheeze, and cough (2). EIB is a specific and common morbidity of childhood asthma and exercise can be used as an indirect provocation test to diagnose asthma and evaluate asthma control (3, 4). A standardized exercise challenge test (ECT) is the reference standard for the diagnosis of EIB (5), but is time-consuming and not accessible for all pediatricians. In daily clinical practice, most pediatricians rely on self- and parent reported respiratory symptoms. However, several studies have shown a poor relation between self-reported symptoms and EIB as measured with an ECT (6–8). Other conditions such as poor cardio-vascular fitness and dysfunctional breathing can influence the perception of asthma symptoms. Nowadays patients can show video images during outpatient clinical visits of symptoms that they experienced at home. A correct assessment of these videos could be a practical alternative for an ECT. It is however unknown whether pediatricians are able to assess the severity of airway obstruction based on these videos. We aimed to study the ability of pediatricians to assess EIB from post-exercise videos.

## Methods

### Patients

This study was part of another study on EIB (9). In this previously published paper based on the same study population of asthmatic children, we describe the prediction of EIB by pediatricians based on information available during a standard outpatient clinic visit. For details on recruitment and in- and exclusion criteria, we refer to this aforementioned paper. A STARD checklist (10) for this study can be found in the Supplementary Material.

### Study Procedure

#### Clinical Assessment and Exercise Challenge Test

Prior to the ECT, a clinical assessment was performed including a medical history with focus on asthma symptoms and a physical examination with pulmonary auscultation.

Spirometry was performed using standard ATS/ERS protocol (11). The ECT was performed in a climate chamber with dry, cold (10.0–12.0°C) air following widely used guidelines (5). For details on spirometry measurements and the ECT, we refer to our other study on EIB (9).

EIB severity was classified by the maximum fall in forced expiratory volume in 1 s (FEV_1_) after exercise (Table 1), as suggested by Anderson et al. (12).

**Table 1:** Classification of EIB severity^a^.

**Degree of EIB severity**	**Maximum fall in FEV_**1**_ after exercise**
No EIB	<10%
Mild EIB	≥10% but <25%
Moderate EIB	≥25% but <50%
Severe EIB	≥50% for steroid-naïve patients
	≥30% for steroid-treated patients

a *EIB severity classification, adopted from Anderson et al. (12)*.

#### Video Recordings and Evaluation by Pediatricians

Video recordings were made using an iPad mini attached to a tripod, positioned to record the patients' head and bare chest (9). Video recordings were made during the clinical assessment before the ECT and shortly after the ECT, before the first post-exercise spirometry measurements.

Twenty pediatricians from three different teaching hospitals (Medisch Spectrum Twente, Isala Zwolle, ZGT Almelo/Hengelo) separately evaluated 5 children that were randomly assigned to them, providing 100 evaluations. First, EIB severity was predicted based on the information from the participants' medical history, physical examination, baseline spirometry results and pre-exercise video images, to provide a general expression of the children. These results are described in a different study by our study group (9).

After this, the post-exercise videos were assessed. The pediatricians scored the videos on the following clinical symptoms of airway obstruction as binary variables: wheezing, nasal flaring, prolonged expiratory phase and jugular and clavicular retractions. Then, pediatricians predicted EIB severity based on a continuous scale ranging from no EIB (0) to very severe EIB (10) and in different EIB severity classifications (Table 1).

### Statistical Analyses

The maximum fall in FEV_1_ (%) was calculated and used for statistical analyses. Results were expressed as mean values ± standard deviation (SD) for normally distributed data and as median ± interquartile range (IQR) for non-normally distributed data. For nominal or ordinal data, numbers with corresponding percentages were used.

Sensitivity was calculated as the proportion of children with EIB, diagnosed with an ECT as reference standard, who were given an EIB diagnosis (mild, moderate, or severe) by the pediatricians. Specificity was calculated as the proportion of children without EIB who were labeled as “no EIB” by the pediatricians. The positive predictive value was calculated as the proportion of children with a pediatricians' predicted EIB diagnosis (mild, moderate, or severe), who actually had EIB (mild, moderate or severe) based on the ECT as reference standard. The negative predictive value was calculated as the proportion of children that were labeled as “no EIB” by the pediatricians and also did not have EIB based on the ECT.

The 95% confidence intervals (CI) for the sensitivity, specificity, positive and negative predictive value were calculated using Episheet (13).

The correlation between predicted EIB severity scored on a continuous rating scale and fall in FEV_1_ after the ECT was calculated with Spearman's rho for non-normally distributed continuous variables. Correlation coefficients were considered as follows: > 0.80 = very strong; 0.60–0.79 = strong; 0.40–0.59 moderate, 0.20–0.39 weak; <0.20 very weak (14). Concordance between the predicted EIB severity classifications and the validated classifications after the ECT was calculated with a linear weighted Cohen's Kappa. Cohen's Kappa values were classified as: <0 = poor; 0–0.2 = slight; 0.2–0.4 = fair; 0.4–0.6 = moderate; 0.6–0.8 = substantial; 0.8–1.0 = almost perfect (15). Sensitivity, specificity and relative risks were calculated for the different clinical symptoms in their relation to EIB, diagnosed with an ECT. A generalized linear model was used to assess the relationship between the different clinical symptoms and fall in FEV_1_ (%). After the univariate relationships were explored, the model was narrowed step by step by excluding non-significant variables until a final model was attained. All 100 evaluations were included for statistical analysis, acknowledging the fact that each child was present multiple times in the dataset, albeit assessed by different pediatricians. A two-sided *p* < 0.05 was considered statistically significant. Data analyses were performed with SPSS® Statistics (version 25.0) and www.vassarstats.net/kappa.html was used to carry out the weighted Cohen's Kappa analyses.

### Ethical Considerations

This study was approved by the Medical Ethics Review Board Twente (identification K15-03) and registered in the Dutch clinical trial register (NTR, identification NL5280).

All children and parents/guardians received written patient information and provided written informed consent before participating in the study.

## Results

Of twenty-four initially included children, three children had spirometry-induced bronchoconstriction and one child had used salbutamol shortly before the ECT. Twenty children completed the protocol and were included for statistical analyses. Twenty pediatricians independently assessed 5 children, providing a total of 100 assessments.

### Characteristics of the Study Population

Baseline characteristics of the included children are shown in Table 2. The mean baseline FEV_1_ was 92.7% of predicted (SD 13.9), with a median fall in FEV_1_ after exercise of 15.1% (IQR 1.2-65.1). Nine children did not have EIB, 4 children had mild EIB, 2 children had moderate and 5 children had severe EIB.

**Table 2:** Characteristics of the study sample (*n* = 20)^a^.

**Variables**	
**Sex**^b^	
Female	10 (50.0%)
Male	10 (50.0%)
Age, years	11.6 (3.4)
BMI, kg/m^2^	19.5 (4.6)
Atopy^b^	11 (55.0%)
FEV_1_ predicted, %	92.7 (13.9)
Fall in ${\text{FEV}}_{1}^{\text{c}}$, %	15.1 (1.2–65.1)
**EIB classification**^b^	
No EIB (<10%)	9 (45.0%)
Mild EIB (10–25%)	4 (20.0%)
Moderate EIB (25–50%)	2 (10.0)
Severe EIB (>50% or ICS use with >30%)	5 (25.0)
Reversibility^c^, %	18.9 (−11.0 −62.3)

a *Values are presented as mean (SD), except when indicated otherwise*.

b Value is presented as n (%).

c *Value is presented as median (IQR). BMI, Body Mass Index (kg/m2); FEV_1_, Forced Expiratory Volume in 1 s; EIB, exercise-induced bronchoconstriction*.

### Prediction of EIB by Pediatricians

**Figure 1:**
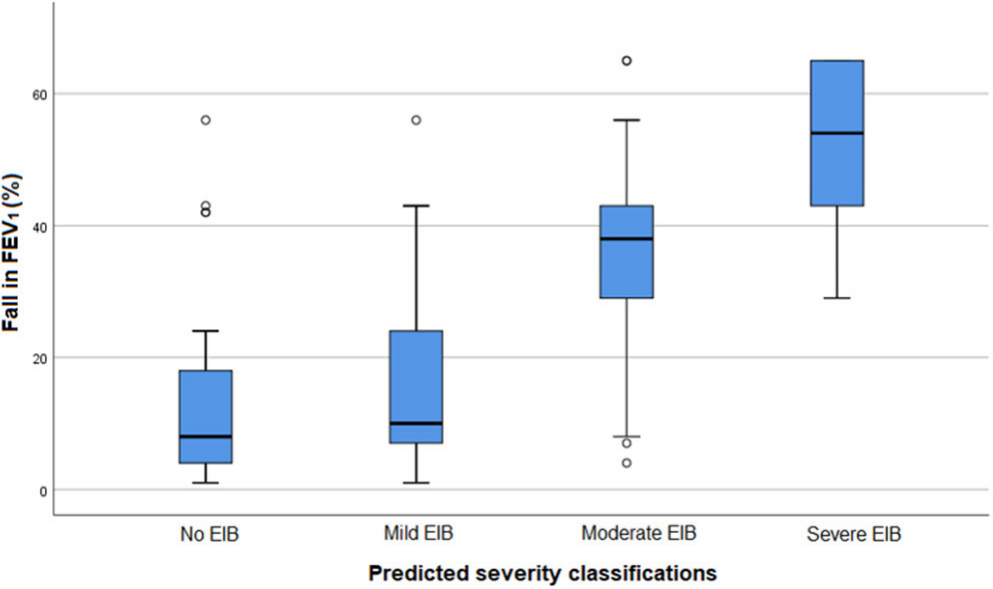
Fall in FEV_1_ after a standardized exercise challenge test and EIB severity as predicted by pediatricians in 100 assessments. FEV_1_, Forced Expiratory Volume in 1 s. EIB: exercise-induced bronchoconstriction.

EIB severity based on the standardized exercise challenge test was compared with EIB severity as predicted by pediatricians, based on the provided clinical information and post-exercise videos (Table 3 and Figure 1).

**Table 3:** Overview of EIB severity as predicted by pediatricians and actual EIB severity measured with an ECT^a^.

	**EIB severity after ECT**					
**EIB severity predicted by pediatricians**		**No EIB**	**Mild EIB**	**Moderate EIB**	**Severe EIB**	**Total**
	No EIB	18	8	3	1	30
	Mild EIB	23	11	6	2	42
	Moderate EIB	4	1	11	6	22
	Severe EIB	0	0	3	3	6
	Total	45	20	23	12	100

a *Predictions from 100 assessments made by 20 different pediatricians based on medical history, physical examination, spirometry result and pre- and post-exercise video. EIB, exercise-induced bronchoconstriction; ECT, exercise challenge test*.

Pediatricians detected EIB with a sensitivity of 78% (95% CI 66–88%) and a specificity of 40% (95% CI 27–54%). The positive predictive value for a pediatricians' diagnosis of EIB was 61% (95% CI 50–72%). The negative predictive value was 60% (95% CI 42–76%).

Participants without EIB or with mild EIB were quite properly assessed, as in only 5 out of 65 assessments the severity was predicted as moderate or severe. In 8 out of 20 children with mild EIB, the severity was underestimated. Children with moderate EIB were overestimated in 3 out of 23 assessments and underestimated in 9 assessments. EIB severity was notably underestimated in participants with severe EIB, with an underestimation in 9 out of 12 assessments.

The agreement between the pediatricians' predicted EIB severity classifications and the validated classifications after the ECT was fair [Kappa = 0.36 (95% CI 0.23–0.48)].

The correlation between the continuous rating scale scores (0–10) and fall in FEV_1_ after the ECT was weak (r_s_ = 0.39, *p* < 0.001).

### Dyspnea Symptoms

The occurrence of dyspnea symptoms after exercise and the sensitivity, specificity and relative risks of these symptoms for EIB are shown in Table 4. Jugular retractions and a prolonged expiratory phase where the most common clinical symptoms in children with EIB, with an occurrence of respectively 64% and 62%. Wheezing had the lowest occurrence (22%) and also the lowest sensitivity for an EIB diagnosis [22% (95% CI 13–35%)] but the highest specificity [93% (95% CI 82–98%)]. Jugular retractions had the highest sensitivity [65% (95% CI 51–76%)] but the lowest specificity [55% (95% CI 40–68%)].

**Table 4:** Occurrence of dyspnea symptoms and their sensitivity, specificity, and relative risk for ElB^a^.

**Symptoms**	**EIB**	**No EIB**	**Sensitivity**	**Specificity**	**RR**
			**(95% CI)**	**(95% CI)**	**(95% CI)**
Wheezing	22% (12/55)	7% (3/45)	22% (13-35)	93% (82–98)	1.6 (1.1–2.2)
Nasal flaring	31% (17/55)	11% (5/45)	31% (20–44)	89% (77–95)	1.6 (1.2–2.2)
Supraclavicular retractions	60% (33/55)	29% (13/45)	61% (48–73)	71% (57–82)	1.8 (1.2–2.6)
Jugular retractions	64% (35/55)	44% (20/45)	65% (51–76)	55% (40–68)	1.4 (1.0–2.1)
Prolonged expiratory phase	62% (34/55)	33% (15/45)	62% (49–73)	67% (52–79)	1.7 (1.2–2.5)

a *Scored by pediatricians in 100 assessments. Sensitivity and specificity of symptoms are for an EIB diagnosis based on the results of a standardized exercise challenge test. EIB, exercise-induced bronchoconstriction; RR, relative risk*.

The relationship between the different clinical symptoms of dyspnea and fall in FEV_1_ after exercise are shown in Table 5. In the univariate analysis, all symptoms were related to fall in FEV_1_. In the multivariate analysis, wheezing, supraclavicular retractions and a prolonged expiratory phase remained independently related to fall in FEV_1_. The presence of wheezing was related to a larger fall in FEV_1_ of 11%. The presence of supraclavicular retractions was related to a larger fall in FEV_1_ of 8.4% and a prolonged expiratory phase with 8.8%. The final prediction model, including these three symptoms, had an explained variance of 25.2% for fall in FEV_1_.

**Table 5:** Clinical dyspnea symptoms and their relation to fall in FEV_1_ (%)^a^.

**Symptoms**	**Univariate analysis**	**Multivariate analysis**
	***B*-value (95% CI)**	***B*-value (95% CI)**
Wheezing	−19.7% (−31.7; −7.7)	**−11.0% (−20.6; −1.4)**
Nasal flaring	−18.2% (−33.9; −2.4)	n.s.
Supraclavicular retractions	−13.7% (−23.6; −3.8)	**−8.4% (−15.5**; **−1.3)**
Jugular retractions	−9.9% (−19.7; −0.7)	n.s.
Prolonged expiratory phase	−15.0% (−25.9; −4.5)	**−8.8% (−16.8**; **−0.8)**

a *Scored by pediatricians in 100 assessments. Fall in FEV_1_ (%) during a standardized exercise challenge test. FEV_1_, Forced Expiratory Volume in 1 s*.

*The bold values are the statistically significant values*.

## Discussion

This study shows that pediatricians have at best a fair ability (Kappa = 0.36) to assess EIB severity from post-exercise videos. EIB severity was especially underestimated in participants with severe EIB (underestimation in 75%). The positive predictive value for a pediatricians' diagnosis of EIB was 61%. The negative predictive value was 60%.

Pediatricians were able to correctly score asthma symptoms on video analyses as wheezing (-11%), supraclavicular retractions (−8.4%) and a prolonged expiratory phase (−8.8%) were found to be independently related to fall in FEV_1_. However, the low occurrence of EIB symptoms seems to compromise an accurate analysis of EIB based on videos.

To our knowledge, this is the first study that focused on the ability of pediatricians to assess EIB severity from post-exercise videos. Related to our study, van Sickle et al. (16) found that there is an important variation in the perception and labeling of asthma symptoms among pediatricians, based on a video questionnaire showing different clinical presentations of asthma. Pediatricians who reported respiratory symptoms from the videos had an 80% higher odds of suggesting an asthma diagnosis compared to pediatricians that did not report these respiratory symptoms. This shows that in order to be able to diagnose asthma, a correct identification of respiratory symptoms is essential. This is in line with our study, as we observed that some physical signs were independently associated with decline in lung function.

The assessment and interpretation of asthma symptoms is not only difficult for pediatricians, but also for children themselves. Baets et al. (7) found that self-reported exercise-induced respiratory symptoms after a free running test had a poor positive predictive value for EIB in school-age children. Seear et al. (6) and Rietveld et al. (8) also showed a poor relation between self-reported symptoms and EIB as measured with an ECT.

Apparently, the interpretation of asthma symptoms during or after exercise, is difficult. Besides recall bias and an overall low disease-perception influencing self-reported symptoms, other conditions such as poor fitness and dysfunctional breathing could influence the perception of asthma symptoms for both children and their caregivers.

In daily clinical practice, wheezing is an important symptom of EIB. In our explorative analysis for the relationship between the different physical symptoms and EIB, wheezing was indeed independently associated with the severity of exercise-induced decline in lung function (11% larger fall in FEV_1_ when wheezing was present). However, we found a low occurrence of wheezing in children with EIB (22%), which is in line with a study by van Leeuwen et al. (17). Even though this low occurrence compromises wheezing as an accurate diagnostic tool to detect EIB, in a population at risk for EIB where post-exercise wheezing is present, a standardized ECT might not be necessary.

Other independently associated symptoms were supraclavicular retractions (−8.4%) and a prolonged expiratory phase (−8.8%). So when an asthmatic individual would present itself with all three symptoms, it is greatly expected that EIB is present, as these symptoms combined predict a fall in FEV_1_ after exercise of 28.2%. However, these symptoms combined had an explained variance of 25.2% for fall in FEV_1_ in our linear model, indicating that the assessment of these symptoms alone is not appropriate for the detection of EIB from videos.

A strength of this study is the execution of the standardized exercise challenge tests. The tests were performed in a cold and dry climate chamber, following standard protocol. Children 6–7 years old performed the test on a jump castle. This method has previously been validated by members of our study group (17). Our study results are subject to several limitations. First, analysis were made with an inflated sample size, as 20 pediatricians each assessed 5 children from a group of 20 children. Although assessed by different pediatricians, each child was presented multiple times in our database. Also, we did not test the accuracy of the assessments of the different physical symptoms by the pediatricians. Because we used subjective assessments for the presence of these symptoms instead of a reference standard, our prediction model might be less precise.

Currently, the monitoring of pediatric asthma consists of infrequent scheduled medical visits and lung function assessments. This strategy cannot fully capture the actual dynamics of the severity of exercise-induced respiratory symptoms and other asthma symptoms, since children are often asymptomatic during these scheduled visits. Video images of putative daily asthma symptoms could provide relevant and easily available information for pediatricians to support the monitoring of asthma control. However, this study shows that pediatricians cannot accurately assess the severity of EIB from post-exercise videos, even when acquired in a standardized environment. It is to be expected that videos from daily situations at home are even more difficult to assess. Future efforts that focus on unraveling the relation of different physical symptoms to the severity of exercise-induced lung function decline in large study groups, could improve the video-assessment of EIB.

In conclusion, this study shows that pediatricians have at best a fair ability to assess EIB severity from post-exercise videos, implicating that standardized ECT's are still vital in the assessment of EIB.

## Transparancy and Contribution Statement

The authors declare that a previously published article by our study group entitled; “*Assessing Exercise-Induced Bronchoconstriction in Children; The Need for Testing”* by Lammers et al. (9), was based on the same study population as this recent article.

In the aforementioned publication we describe that the prediction of EIB occurrence and severity by pediatricians based on information that is available during a standard outpatient clinic visit (medical history, physical examination, baseline spirometry results and pre-exercise video images for an overall impression of the patient), was poor.

In this present manuscript we describe the ability of pediatricians to assess EIB and physical symptoms of EIB from post-exercise videos, filmed after a standardized exercise challenge test.

## Data Availability Statement

The datasets generated for this study are available on request to the corresponding author.

## Ethics Statement

The studies involving human participants were reviewed and approved by METC Twente. Written informed consent to participate in this study was provided by the participants' legal guardian/next of kin.

## Author Contributions

All authors contributed equally on the writing and editing of the research protocol and manuscript. NL, MH, RS-V, JD, and BT contributed to data acquisition. NL, MB-K, BT, and JP contributed to the data analysis.

### Conflict of Interest

The authors declare that the research was conducted in the absence of any commercial or financial relationships that could be construed as a potential conflict of interest.
